# Paternal care and litter size coevolution in mammals

**DOI:** 10.1098/rspb.2016.0140

**Published:** 2016-04-27

**Authors:** Paula Stockley, Liane Hobson

**Affiliations:** Mammalian Behaviour and Evolution Group, Institute of Integrative Biology, University of Liverpool, Leahurst Campus, Chester High Road, Neston CH64 7TE, UK

**Keywords:** biparental care, life history, litter size, paternal care, mammals, monogamy

## Abstract

Biparental care of offspring occurs in diverse mammalian genera and is particularly common among species with socially monogamous mating systems. Despite numerous well-documented examples, however, the evolutionary causes and consequences of paternal care in mammals are not well understood. Here, we investigate the evolution of paternal care in relation to offspring production. Using comparative analyses to test for evidence of evolutionary associations between male care and life-history traits, we explore if biparental care is likely to have evolved because of the importance of male care to offspring survival, or if evolutionary increases in offspring production are likely to result from the evolution of biparental care. Overall, we find no evidence that paternal care has evolved in response to benefits of supporting females to rear particularly costly large offspring or litters. Rather, our findings suggest that increases in offspring production are more likely to follow the evolution of paternal care, specifically where males contribute depreciable investment such as provisioning young. Through coevolution with litter size, we conclude that paternal care in mammals is likely to play an important role in stabilizing monogamous mating systems and could ultimately promote the evolution of complex social behaviours.

## 1. Introduction

Parental care was identified by Darwin [1] as the foundation of complex social behaviour in vertebrates and is central to the biology of birds and mammals. In both groups, offspring are routinely nourished, kept warm and protected by one or more parents, and the number successfully reared is strictly limited by the high costs of caring for them [2–4]. Although parents of either sex can potentially contribute to these costs, care by males is relatively uncommon among mammals [5]. To some extent, this may be explained by the specialized adaptations of gestation and lactation characteristic of female mammals. Such extreme specialization of maternal care means females are highly adapted for parental investment, whereas males are physically dissociated from offspring during early development [2,6]. Nonetheless, male mammals can provide parental care, sometimes intensively so, with known examples in around 10% of mammalian genera [5] and 59% of socially monogamous species [7]. Despite numerous well-documented examples, though, the evolutionary causes and consequences of paternal care in mammals are not well understood.

To understand the evolution of parental care requires consideration of both the potential benefits and costs involved [2,6,8]. A key cost of parental care for males is a likely trade-off with investment in pursuing additional mating opportunities [6,9,10]. Hence male care should only evolve if the costs of any lost mating opportunities are outweighed by the fitness benefits of caring. Potential fitness benefits of paternal care include effects on both the number and quality of offspring reared. Male care can lead to increased offspring survival rates [11–13], and may be particularly beneficial in promoting offspring survival when clutch or litter size is large [14,15]. Paternal care has also been reported to influence offspring quality, resulting in the production of larger or faster developing offspring [16–19]. Again, such benefits may be particularly valuable where costs of offspring production are high, for example if neonate mass is large relative to female body mass [20,21]. Male care might therefore be more likely to evolve among species where females produce relatively large litters or large neonates for their body size. Importantly though, once male care evolves, it may allow females to increase litter size [15] or offspring size, and/or to produce young at a faster rate [7,22]. Coevolution of paternal care and life-history traits may therefore make it difficult to assess whether biparental care has evolved because of the importance of male care to offspring production.

In this study, we investigate the evolution of paternal care and life-history traits related to offspring production in mammals. We first explore if paternal care is associated with relatively high rates of offspring production (larger litter size, more offspring per teat, shorter inter-litter intervals, higher annual fecundity), or larger offspring relative to adult body size (larger neonate or weaning mass), each of which might be a potential evolutionary cause or consequence of male investment. To further investigate significant associations between paternal care and offspring production traits, we then perform phylogenetic reconstructions of the pattern of evolutionary transitions between correlated traits. Our analyses allow us to explore if biparental care is likely to have evolved because of the importance of male care to offspring production, and/or if evolutionary increases in offspring production result from the evolution of paternal care.

## 2. Material and methods

### (a) Data collection

Data on paternal care were collated from published reviews and a systematic search of the primary literature using Web of Science (electronic supplementary material, table S1). Paternal care behaviours in mammals are typically split into broad categories of direct and indirect care for offspring [2,5,11,23,24]. Here, we adopt a conservative definition of paternal care, including only those activities likely to be of most direct benefit to offspring. We classify paternal care as present for species in our dataset if males are reported to provide food, huddle and sleep with young, retrieve, carry, groom or clean them, babysit, and/or actively defend young from predators or conspecifics. Notably, certain direct forms of care are depreciable, such that investment in one individual or litter precludes investment in others [2,5]. Depreciable forms of male care such as provisioning (directly to the offspring, or via the mother during offspring development) may be particularly beneficial in relaxing energetic constraints on offspring production for females. We therefore identify separately those species in which males are reported to provide food, in order to test for evidence of increased reproductive output. We also adopt a conservative approach in classifying species with an absence of paternal care behaviour, including only species in our dataset for which an absence of paternal care is stated in cited references (electronic supplementary material, table S1). Where male care behaviour is reported as present but infrequent or occasional, we adopt a classification of no care. Where sources provide conflicting accounts of male involvement in offspring care, we have given priority to evidence of behaviour in natural or semi-natural environments where possible. In total, we collated data for 427 mammalian species, with 119 classified as having paternal care (within orders Afrosoricida, Artiodactyla, Carnivora, Chiroptera, Diprotodontia, Macroscelidea, Primates, Rodentia, Soricomorpha), and 48 as having paternal care that includes provisioning of young (further details in electronic supplementary material, table S1).

To investigate relationships between paternal care and the number and size of offspring produced, we collected information for species in our dataset on average litter size, offspring number per teat, neonate mass, weaning mass, inter-litter interval and number of litters per year (calculating annual fecundity as the product of average litter size and number of litters per year). Since life-history traits are closely linked to body size, we also collected data for average adult body mass to include as a covariate in our statistical analyses. Data were obtained by cross-referencing between published reviews and databases [25–30], with means taken when multiple literature sources were available for a given species. For offspring to teat ratios, where sources produced conflicting average values (e.g. cross-referencing between Hayssen *et al.* [25] and Jones *et al.* [30]), we used the value closest to 0.5 (see below).

### (b) Comparative analyses

Species in comparative analyses cannot be considered as statistically independent, because they share common ancestry; hence it is important to take into account phylogenetic relatedness [31,32]. To control for potential statistical non-independence of species, we used comparative methods incorporating phylogenetic information. We used the best estimate dated mammalian supertree of Fritz *et al.* [33], pruned to match species in our dataset using *ape* [34] and *geiger* [35] packages in the statistical program R (v. 3.0.2; [36]).

Continuous variables were log transformed prior to analysis to reduce skew. However, litter size in mammals has a bimodal distribution, with species either typically producing one or multiple offspring per reproductive event. We therefore conducted separate analyses for polytocous and monotocous species, both to meet assumptions of parametric statistical analyses, and to explore relevant life-history traits separately for each category (e.g. paternal care might be more important to offspring size among species that typically produce a single offspring per reproductive attempt). We also conducted analyses for monotocous and polytocous species combined, using data on offspring number per teat as a measure of offspring production. According to ‘the one half rule’ for mammals, average litter size is typically half the number of teats available [37–39]. Since teat number is relatively evolutionarily conserved compared to litter size [37], species that break the one half rule by producing relatively more offspring per teat are likely to have increased their average litter size rather than decreased their number of teats [39]. Importantly, as offspring to teat ratios of around 0.5 are commonly found in a broad range of mammals (electronic supplementary material, figure S1), this continuous variable allows us to explore variation in offspring production for all species in our dataset combined.

We used a phylogenetic generalized least-squares (PGLS) modelling approach, performed using *caper* [40] in R (v. 3.0.2 [36]), to test for hypothesized evolutionary relationships between paternal care and life-history traits. This takes into account potential non-independence of species in the dataset based on the phylogeny and a maximum-likelihood estimate of the phylogenetic scaling parameter *λ*, varying between 1 (indicating a strong phylogenetic signal) and 0 (indicating no phylogenetic signal). Paternal care was categorized as 0 (absent) or 1 (present), and adult body mass was included as a covariate in all models. We used a PGLS approach to test for evidence that paternal care (or paternal care that includes provisioning) is associated with high levels of offspring production (larger litter size, more offspring per teat, shorter inter-litter intervals, higher annual fecundity) or large offspring size (larger neonate mass, larger weaning mass), as predicted if paternal care increases male reproductive success via increased offspring numbers or quality, respectively. To test the robustness of our results and rule out potential confounding effects of other forms of extra-maternal care on offspring production, we conducted additional analyses excluding species with cooperative breeding. Cooperatively breeding species were identified as those in which non-breeding females contribute to provisioning or carrying young born to other females, following Lukas & Clutton-Brock [41].

We used maximum-likelihood methods within the module discrete [42] of BayesTraits [43] to test for evidence of correlated evolution between categorical traits. The tests involve analysis of relationships between two binary traits; in this case, the presence or the absence of paternal care, and the presence or the absence of relatively large litter size. Paternal care data were split into two binary traits: (i) the presence or the absence of paternal care (including all instances of care versus no care) and (ii) the presence or the absence of male provisioning (paternal care with provisioning, versus paternal care without provisioning or no paternal care). To classify species as having a relatively large litter size or not, we split species in our dataset according to: (i) whether they typically produce multiple offspring (polytcocy) or a single offspring (monotocy) per reproductive event and (ii) whether or not they produce more offspring than expected under the one half rule for mammals [37,38]. Based on the distribution of offspring: teat ratios for species in our dataset (electronic supplementary material, figure S2), we classed species as having relatively large litters for their teat number if their average number of offspring per teat was greater than or equal to 0.55. This includes the upper 45% range of offspring to teat ratios for species in our dataset. To maximize sample sizes, we ran analyses using all species for which data were available for at least one trait of interest. Where trait values were unknown we specified that the trait could occur in either state [42]. We investigated evidence of correlated evolution by first reconstructing the evolution of paternal care and litter size according to the most likely scenario if each of the two traits evolved entirely independently within the phylogenetic tree, and compared this with a scenario in which they are correlated and evolutionary transitions occur between four possible combinations (no paternal care and relatively small litter size, no paternal care and relatively large litter size, paternal care and relatively small litter size, paternal care and relatively large litter size). We used the best estimate supertree of Fritz *et al*. [33] to control for phylogeny, with polytomies randomly resolved using *ape* [34]. Likelihoods were estimated using 10 optimization attempts per run, and statistical significance was assessed using likelihood ratio tests, comparing twice the difference between the independent and dependent models using a *χ*^2^-test with four degrees of freedom.

To model the evolution of correlated traits, we used a likelihood framework and Bayesian inference, using a Markov chain Monte Carlo (MCMC) sampling algorithm and reversible jump (RJ) procedure in BayesTraits [42,43]. This approach allowed us to derive Bayesian posterior distributions of model log likelihoods, transition rate parameters, and inferred ancestral states within the mammal phylogeny. We used a sample of 100 resolved trees [33] to construct the models. An exponential prior distribution was used, as our maximum-likelihood tests suggested that transition rates (the frequency of trait changes per unit branch length) were low [44]. The prior was seeded from a range of 0–2, and the rate deviation value was approximated using Autotune within BayesTraits V2 [42,43]. Each MCMC chain was run five times, for 1 000 000 iterations sampled every 1000, with the first 100 000 excluded as a burn-in period, to ensure that convergence had been reached. Convergence was assessed visually using Tracer v. 1.6 [45]. Results are reported for the run with the median harmonic mean value, taken from the post-convergence portion of the five runs. Post-convergence iterations were used to calculate how likely a transition between states was to occur (using the *Z*-score) and the most likely combination of relative transition rate values. Transition rates with higher *Z*-scores were considered less likely to occur, while transition rate combinations were considered more likely to occur the more frequently they were observed. For more information on transition rate combinations, see electronic supplementary material, table S2.

## 3. Results

### (a) Associations between paternal care and offspring production

**Figure 1.**
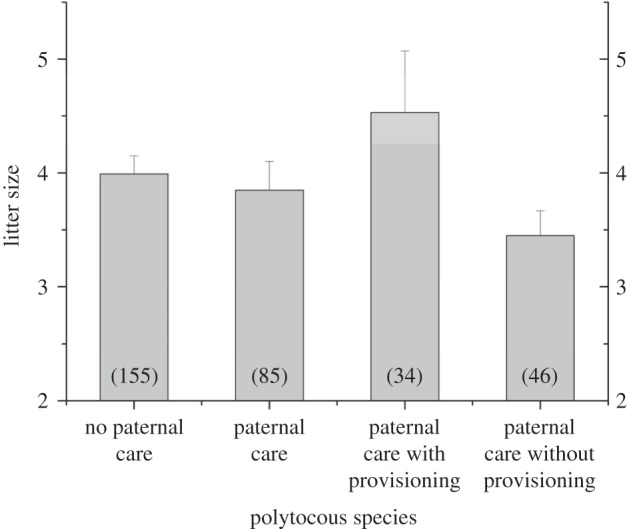
Average litter size (mean ± s.e.) for polytocous mammals with contrasting levels and types of paternal care. Sample sizes are shown in parentheses. Statistical analyses of litter size variation with control for phylogeny and body mass are provided in table 1*a*.

We first used a PGLS approach to investigate whether variation in litter size among polytocous species is related to paternal care. Contrary to the prediction that male care should be associated with high levels of offspring production, we found no evidence overall that polytocous species with paternal care have significantly larger average litter sizes (table 1*a*(i) and figure 1; mean ± s.e. litter size for species with paternal care = 3.86 ± 0.25 (*n* = 85), and without paternal care = 3.99 ± 0.16 (*n* = 155)). However, when considering only those species for which males provide food, paternal care is significantly associated with larger litter sizes (table 1*a*(ii) and figure 1; mean ± s.e. litter size for polytocous species with paternal provisioning = 4.53 ± 0.54 (*n* = 34), compared to paternal care without provisioning = 3.45 ± 0.22 (*n* = 46), and no paternal care = 3.99 ± 0.16 (*n* = 155)). Similar patterns are found in relation to average numbers of offspring produced per teat for both polytocous and monotocous species (table 1*b*): there is no association when considering all species with paternal care (table 1*b*(i)), but species with paternal provisioning produce significantly more offspring per teat (table 1*b*(ii)). A significant positive relationship between paternal provisioning and litter size, and a strong trend for species with paternal provisioning to produce more offspring per teat, was also found in analyses excluding cooperatively breeding species (electronic supplementary material, table S3). Our findings thus reveal that male care with provisioning is associated with relatively large litter sizes among polytocous species, both absolutely and relative to teat number.

**Table 1.** Phylogenetically controlled PGLS models of the relationships between paternal care and offspring production in mammals. Models test for relationships with average: (*a*) litter size, (*b*) offspring number per teat, (*c*) inter-litter interval and (*d*) annual fecundity of females, linked to: (i) paternal care and (ii) paternal care that includes provisioning of offspring. Litter size is for polytocous species only. Body mass is included as a covariate in all models. Significant values (*p* < 0.05) are presented in bold text. For the phylogenetic scaling parameter *λ*, superscripts indicate if values are significantly different from 0 or 1, respectively (where n.s. = not significantly different, and * = significantly different at *p* < 0.05) in likelihood ratio tests.

trait		*λ*	d.f.	predictor	slope ± se	*t*	*p*-value
(*a*) litter size	(i) paternal care	0.88^*,*^	230	body mass	−**0.04 ± 0.02**	−**2.41**	<**0.02**
				paternal care	−0.01 ± 0.02	−0.51	0.62
	(ii) paternal provisioning	0.89^*,*^	225	body mass	−**0.04 ± 0.02**	−**2.30**	<**0.03**
				paternal provisioning	**0.09 ± 0.03**	**2.82**	<**0.006**
(*b*) offspring per teat	(i) paternal care	0.93^*,*^	222	body mass	−**0.06 ± 0.02**	−**2.94**	<**0.004**
				paternal care	−0.01 ± 0.03	−0.30	0.77
	(ii) paternal provisioning	0.90^*,*^	217	body mass	−**0.06 ± 0.02**	−**3.19**	<**0.002**
				paternal provisioning	**0.12 ± 0.04**	**3.25**	<**0.002**
(*c*) inter-litter interval	(i) paternal care	0.98^*,n.s.^	269	body mass	**0.13 ± 0.02**	**6.74**	<**0.001**
				paternal care	0.02 ± 0.03	0.63	0.53
	(ii) paternal provisioning	0.98^*,n.s.^	261	body mass	**0.13 ± 0.02**	**6.56**	<**0.001**
				paternal provisioning	−0.019 ± 0.03	−0.59	0.56
(*d*) annual fecundity	(i) paternal care	0.92^*,*^	219	body mass	−**0.10 ± 0.02**	−**4.61**	<**0.001**
				paternal care	0.04 ± 0.03	1.27	0.21
	(ii) paternal provisioning	0.94^*,*^	217	body mass	−**0.10 ± 0.02**	−**4.87**	<**0.001**
				paternal provisioning	**0.16 ± 0.04**	**3.50**	<**0.001**

We next used maximum-likelihood tests within discrete [46] to investigate whether paternal care is evolutionarily correlated with the production of multiple offspring per reproductive attempt (polytocy), or with large litter size relative to teat number when classed as a binary trait. As described above, male care was classified according to: (i) the presence or the absence of paternal care (including all instances of care versus no care) and (ii) the presence or the absence of male provisioning (paternal care with provisioning, versus no paternal care or paternal care without provisioning). Based on a comparison of dependent and independent models, we found no evidence for dependent evolution of paternal care and polytocy (log-likelihood test: dependent model = −286.4, independent model = −288.4, *n* = 427, d.f. = 4, ML test = 4.01, *p* = 0.40), and no evidence for dependent evolution of paternal care with provisioning and polytocy (log-likelihood test: dependent model = −208.6, independent model = −206.3, *n* = 427, d.f. = 4, ML test = 4.64, *p* = 0.32). Hence the evolution of paternal care is not significantly correlated with the production of multiple rather than single offspring for species in our dataset. However, consistent with the results of our PGLS analyses (table 1), when classifying offspring production as a binary trait we again found evidence of correlated evolution between paternal care with provisioning and large litter size relative to teat number (log-likelihood test: dependent model = −246.3, independent model = −266.3, *n* = 420, d.f. = 4, ML test = 39.90, *p* < 0.001).

In relation to offspring production rates, results of PGLS analyses indicate that neither paternal care (all types) nor paternal care that includes provisioning is significantly associated with average inter-litter interval for species in our dataset (table 1*c*), suggesting that paternal care does not facilitate more frequent reproduction by females. Consistent with our findings for litter size, there is no significant relationship with average annual fecundity for all species with paternal care (table 1*d*(i)), but species in which males provision their young have significantly higher annual fecundity (table 1*d*(ii)).

### (b) Associations between paternal care and offspring size

We found no evidence that either polytocous or monotocous species with paternal care produce significantly larger offspring at birth or weaning (table 2); hence paternal care is not associated with the production of larger offspring, even with male provisioning when a single offspring is reared.

**Table 2.** Phylogenetically controlled PGLS models of the relationships between paternal care and offspring size in polytocous and monotocous mammals, respectively. Models test for relationships with neonate mass (*a,b*) and weaning mass (*c,d*), linked to: (i) paternal care and (ii) paternal care that includes provisioning of offspring. Body mass is included as a covariate in all models, and litter size is included for models for polytocous species that include paternal care with male provisioning, as larger litter size associated with male provisioning in this group may negatively influence offspring size. Data for neonate mass are for eutherian mammals only. Significant values (*p* < 0.05) are presented in bold text. For the phylogenetic scaling parameter ***λ***, superscripts indicate if values are significantly different from 0 or 1, respectively (where n.s. = not significantly different, and * = significantly different at *p* < 0.05).

trait		*λ*	d.f.	predictor	slope ± se	*t*	*p*-value
(*a*) neonate mass (polytocous)	(i) paternal care	0.99^*,n.s.^	160	body mass	**0.64 ± 0.02**	**28.70**	<**0.001**
				paternal care	−0.001 ± 0.02	−0.02	0.99
	(ii) paternal provisioning	1.00^*,n.s.^	155	body mass	**0.65 ± 0.02**	**29.66**	<**0.001**
				litter size	−**0.18 ± 0.06**	−**3.16**	<**0.002**
				paternal provisioning	−0.02 ± 0.01	−1.23	0.22
(*b*) neonate mass (monotocous)	(i) paternal care	0.87^*,*^	81	body mass	**0.76 ± 0.02**	**30.73**	<**0.001**
				paternal care	−0.01 ± 0.03	−0.27	0.79
	(ii) paternal provisioning	0.86^*,*^	77	body mass	**0.76 ± 0.03**	**29.94**	<**0.001**
				paternal provisioning	−0.004 ± 0.04	−0.10	0.92
(*c*) weaning mass (polytocous)	(i) paternal care	0.26^*,*^	108	body mass	**0.77 ± 0.03**	**29.26**	<**0.001**
				paternal care	0.03 ± 0.06	0.59	0.55
	(ii) paternal provisioning	0.13^*,*^	106	body mass	**0.75 ± 0.03**	**29.88**	<**0.001**
				litter size	−**0.34 ± 0.12**	−**2.80**	<**0.007**
				paternal provisioning	0.09 ± 0.07	1.17	0.25
(*d*) weaning mass (monotocous)	(i) paternal care	0.52^*,*^	71	body mass	**0.88 ± 0.03**	**32.5**	<**0.001**
				paternal care	0.03 ± 0.05	0.66	0.51
	(ii) paternal provisioning	0.54^*,*^	69	body mass	**0.88 ± 0.03**	**32.17**	<**0.001**
				paternal provisioning	−0.04 ± 0.06	−0.60	0.55

### (c) Modelling trait evolution

**Figure 2.**
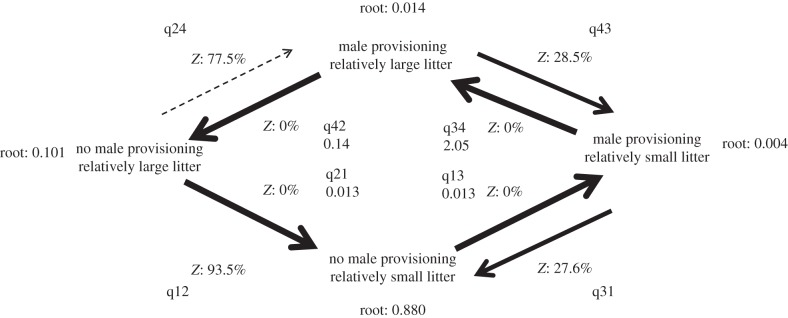
Coevolution between male care that includes provisioning and large litter size relative to teat number in mammals. Species with large litters relative to teat number are identified in relation to the ‘one half rule’, with offspring to teat ratios of 0.55 or above (see main text for further details). Ancestral state reconstructions are shown as root values, which indicate the proportion of the post-convergence portion of the model for different states. Transitions can occur between four states: male care that includes provisioning and the production of relatively large litters; male care that includes provisioning and the production of relatively small litters; no male care and the production of relatively large litters; no male care and the production of relatively small litters. Transition rate names are depicted as *q*_(*xy*)_. *Z*-values reflect the proportion of visits assigned as zero in the post-convergence portion of the model (i.e. lower *Z*-values indicate a higher probability of transition between states). Arrows representing transitions between states are scaled to represent the probability of a transition (thicker lines indicate a lower *Z* value and higher transition probability). *Z*-values more than 50% are represented by dashed lines and *Z*-values more than 90% have no lines. Values below transition rate names (*q*_(*xy*)_) are mean transition rates where *Z* is less than 25%.

Finally, since offspring production is significantly associated with paternal provisioning in our PGLS analyses and likelihood ratio tests, we conducted further analyses to investigate the pattern of evolutionary transitions for these traits in discrete [42], using an MCMC sampling algorithm and RJ procedure. If male care with provisioning facilitates evolutionary increases in litter size, we predict that the evolution of male provisioning will often precede increases in litter size. Conversely, if the production of large or costly litters favours the evolution of male provisioning, we predict that the evolution of larger litter sizes will typically precede transitions to male provisioning. The results presented in figure 2 support the former hypothesis. Ancestral state reconstructions suggest that the most likely ancestral state is one of no male provisioning and an offspring to teat ratio of less than 0.55 (results shown as root values in figure 2).

Transition rate probabilities presented in figure 2 indicate that male provisioning behaviour is more likely to change first (transition rate q13 compared to q12), with a gain in provisioning more likely to occur than an increase in litter size relative to teat number from the ancestral state. Figure 2 further suggests that offspring number per teat is more likely to increase in the presence of male provisioning (transition rate q34) than in its absence (transition rate q12). Hence our analyses indicate that paternal care with provisioning tends to favour the evolution of increased litter size relative to teat number.

## 4. Discussion

It is generally accepted that males should only care for their offspring if the benefits of increased offspring survival outweigh the costs of lost mating opportunities [2,6,8]. Here we find no evidence that mammalian paternal care is more likely to evolve where females produce large offspring or large litters relative to adult body mass. Clearly, male care might still have beneficial effects for offspring survival that is not captured by variation in the life-history traits analysed here [11–14]. But notwithstanding the limitations of this approach, male care is not more common under conditions where additional investment would appear particularly beneficial to females. Moreover, although paternal care with provisioning is associated with relatively large litter sizes, the production of large litters in this case appears more likely to be an evolutionary consequence of male investment rather than a stimulus for the evolution of paternal care. These findings complement previous studies indicating that social monogamy is unlikely to have evolved because biparental care is important for offspring survival [7,44,47]. Instead, the intensity of male care has been shown to correlate negatively with extra-pair paternity rates across 15 socially monogamous mammalian species [48]. However, as yet, it is unclear whether paternity certainty may promote male care [49], or if male care instead enhances the monopolization of females and promotes the paternity success of caring males under competitive conditions [50,51]. Male care is also found in species with polygynous or promiscuous mating systems, and in some cases might even be regarded as a form of mating competition, with care directed to unrelated offspring ([52,53], but see [54]). Further investigation is thus needed to better understand the origins and distribution of male care in mammals with diverse mating systems, including the question of why biparental care is generally uncommon in mammals, when it could be beneficial to offspring production and/or survival. Opportunities for direct male investment might be constrained to species where females invest in offspring through behaviours other than suckling (e.g. carrying or huddling offspring, providing solid food). Nonetheless, despite having the opportunity to do so, relatively few male primates or bats share the prolonged burden of carrying the young, and large numbers of carnivores do not share food with their offspring [5].

Our finding that offspring production is more likely to increase where paternal care includes provisioning is consistent with evidence that female mammals are physiologically constrained in their reproductive investment [55,56]. Life-history trade-offs mediated by such constraints are expected to limit the number and quality of offspring that females are able to produce, as well as their current and future reproductive success [57,58]. Depreciable forms of paternal care such as provisioning could relax constraints on offspring production; for example, an increase in litter size may result from male care allowing females to lower their investment in each individual offspring. Although we have focused on benefits of male provisioning, it is important to emphasize that other forms of depreciable care could also contribute to increases in litter size, and may often occur in association with provisioning. For example, offspring carrying by male callitrichid primates is likely to be particularly important in allowing females to increase the number of offspring reared simultaneously [59,60].

Our analyses also reveal that male provisioning is associated with higher average annual female fecundity. This effect appears to be largely driven by increases in litter size rather than reductions in inter-litter intervals among species in our dataset. We note however that relatively high reproductive rates have previously been reported among socially monogamous mammals with biparental care, in which females produce more litters per year compared with socially monogamous species without biparental care [7]. More generally, the benefits of male care and relationships with offspring production rates are likely to differ according to species life histories and mating systems. For example, huddling behaviour is a common form of paternal care among rodent species with altricial young, and may be most beneficial to offspring survival when litter size is relatively small [61]. Relationships between paternal care and litter size or other life-history traits may therefore be obscured among diverse mammalian groups unless different forms of care and their potential benefits are distinguished.

We found no evidence that paternal care is associated with the production of relatively large offspring, even among monotocous species where parents invest their efforts into sequential rearing of individual young. Whether biparental care should be expected to result in larger or higher quality offspring is debatable however, in part because sexual conflict can have effects on the amount of care that each offspring receives [62–64]. For example, experimental evidence in zebra finches reveals that offspring receive greater *per capita* parental investment from single females than from both parents working together [65]. Our results suggest that females use depreciable male care to produce more offspring rather than to increase offspring size. Similarly, according to the Smith–Fretwell model of optimal offspring size [66], the optimal amount of provisioning per offspring under uni-parental care is independent of the total resource available to a female. So, for example, if the total resources available for offspring production were to double, a female is expected to double the number of offspring produced rather than give twice the amount of care to each one.

Finally, our findings provide new insights regarding the evolutionary stability of mammalian mating systems. Previous studies have confirmed strong associations between monogamy and paternal care in mammals, with the evolution of male care often following the establishment of monogamous mating systems [7,44,47]. Moreover, Opie *et al.* [44] report that once paternal care evolves within monogamy, it is unlikely to be lost. By showing that paternal care can lead to an increase in offspring production, our findings provide a potential explanation for its stability within monogamous mating systems. That is, once established, paternal care should be relatively stable because resultant increases in litter size will make offspring survival more strongly reliant on biparental care. Increased offspring production should thus increase the benefits for males of helping to rear offspring relative to searching for additional mates, and hence stabilize monogamous mating systems [15]. Since cooperative breeding in mammals has also been associated with monogamous mating systems, and with the production of multiple offspring per reproductive attempt [41,67], it is possible that paternal care might also facilitate the evolution of further help in rearing offspring. That is, an increase in offspring production afforded by paternal care might in turn increase the benefits to related helpers of assisting to rear young (and/or the benefits to parents of soliciting help), thereby facilitating further increases in reproductive rate. Thus, consistent with Darwin's [1] original insights, the evolutionary consequences of parental care by male mammals appear significant, with potential ultimately to influence life-history traits, the stability of mating systems and the evolution of complex social behaviours.

## Supplementary Material

Stockley&Hobson_Supplementary Materials

## Supplementary Material

Stockley&Hobson_Supplementary Materials

## Data accessibility

The full dataset supporting this article is available from the Dryad Digital Repository at http://dx.doi.org/10.5061/dryad.5d4fh.

## References

[1] Darwin C 1871 The descent of man and selection in relation to sex. London, UK: John Murray.

[2] Clutton-Brock TH 1991 The evolution of parental care. Princeton, NJ: Princeton University Press.

[3] Farmer CG 2000 Parental care: the key to understanding endothermy and other convergent features in birds and mammals. Am. Nat. 155, 326–334. (10.1086/303323) 10718729

[4] Royle NJ, Smiseth PT, Kolliker M 2012 The evolution of parental care. Oxford, UK: Oxford University Press.

[5] Kleiman DG, Malcolm JR 1981 The evolution of male parental investment. In Parental care in mammals (eds Gubernick DJ, Klopfer PH), pp. 347–387. New York, NY: Plenum.

[6] Maynard-Smith J 1977 Parental investment: a prospective analysis. Anim. Behav. 25, 1–9. (10.1016/0003-3472(77)90062-8)

[7] Lukas D, Clutton-Brock TH 2013 The evolution of social monogamy in mammals. Science 341, 526–530. (10.1126/science.1238677) 23896459

[8] Klug H, Alonzo SH, Bonsall MB 2012 Theoretical foundations of parental care. In The evolution of parental care (eds Royle NJ, Smiseth PT, Kolliker M), pp. 21–39. Oxford, UK: Oxford University Press.

[9] Wade MJ, Shuster SM 2002 The evolution of parental care in the context of sexual selection: a critical reassessment of parental investment theory. Am. Nat. 160, 285–292. (10.1086/341520) 18707439

[10] Magrath MJL, Komdeur J 2003 Is male care compromised by additional mating opportunity? Trends Ecol. Evol. 18, 424–430. (10.1016/S0169-5347(03)00124-1)

[11] Elwood RW 1983 Parental behaviour of rodents. Chichester, UK: John Wiley & Sons Ltd.

[12] Gubernick DJ, Teferi T 2000 Adapative significance of male parental care in a monogamous mammal. Proc. R. Soc. Lond. *B* 267, 147–150. (10.1098/rspb.2000.0979) PMC1690505 10687819

[13] Wright HWY 2006 Paternal den attendance is the best predictor of offspring survival in the socially monogamous bat-eared fox. Anim. Behav. 71, 503–510. (10.1016/j.anbehav.2005.03.043)

[14] Davies NB, Hatchwell BJ 1992 The value of male parental care and its influence on reproductive allocation by male and female dunnocks. J. Anim. Ecol. 61, 259–272. (10.2307/5319)

[15] Smith HG, Härdling R 2000 Clutch size evolution under sexual conflict enhances the stability of mating systems. Proc. R. Soc. Lond. B 267, 2163–2170. (10.1098/rspb.2000.1264)

[16] Dudley D 1974 Contributions of paternal care to the growth and development of the young in *Peromyscus californicus**.* Behav. Biol. 11, 155–166. (10.1016/S0091-6773(74)90305-8) 4847518

[17] Elwood RW, Broom DM 1978 The influence of litter size and parental behaviour on the development of Mongolian gerbil pups. Anim. Behav. 26, 438–454. (10.1016/0003-3472(78)90061-1)

[18] Huber S, Millesi E, Dittami JP 2002 Paternal effort and its relation to mating success in the European ground squirrel. Anim. Behav. 63, 157–164. (10.1006/anbe.2001.1898)

[19] Wright SL, Brown RE 2002 The importance of paternal care on pup survival and pup growth in *Peromyscus californicus* when required to work for food. Behav. Proc. 60, 41–52. (10.1016/S0376-6357(02)00101-8) 12429391

[20] Kleiman DG 1977 Monogamy in mammals. Q. Rev. Biol. 52, 39–69. (10.1086/409721) 857268

[21] Wright PC 1990 Patterns of paternal care in primates. Int. J. Primatol. 11, 89–101. (10.1007/BF02192783)

[22] Cantoni D, Brown RE 1997 Paternal investment and reproductive success in the California mouse, *Peromyscus californicus*. Anim. Behav. 54, 377–386. (10.1006/anbe.1996.0583) 9268470

[23] Dewsbury DA 1985 Paternal behaviour in rodents. Am. Zool. 25, 841–852. (10.1093/icb/25.3.841)

[24] Rosenblatt JS, Snowden CT 1996 Parental care: evolution, mechanism, and adaptive significance. San Diego, CA: Academic Press.

[25] Hayssen V, Tienhoven A, Tienhoven A 1993 Asdell’*s patterns of mammalian reproduction* Ithaca, NY: Cornell University Press.

[26] Silva-Aliaga M, Downing JA 1995 CRC handbook of mammalian body masses. Oxford, UK: CRC Press.

[27] Ewer RF 1998 The carnivores. Ithaca, NY: Cornell University Press.

[28] Nowak RM 1999 Walker’*s mammals of the world* Baltimore, MD: The Johns Hopkins University Press.

[29] Symonds MRE 1999 Life histories of the Insectivora: the role of phylogeny, metabolism and sex differences. J. Zool. Lond. 249, 315–337. (10.1111/j.1469-7998.1999.tb00768.x)

[30] Jones KE et al. 2009 PanTHERIA: a species-level database of life-history, ecology and geography of extant and recently extinct mammals. Ecology 90, 2648 (10.1890/08-1494.1)

[31] Felsenstein J 1985 Phylogenies and the comparative method. Am. Nat. 125, 1–15. (10.1086/284325)

[32] Harvey PH, Pagel MD 1991 The comparative method in evolutionary biology. Oxford, UK: Oxford University Press.

[33] Fritz SA, Bininda-Emonds ORP, Purvis A 2009 Geographical variation in predictors of mammalian extinction risk: big is bad, but only in the tropics. Ecol. Lett. 12, 538–549. (10.1111/j.1461-0248.2009.01307.x) 19392714

[34] Paradis E, Claude J, Strimmer K 2004 APE: analyses of phylogenetics and evolution in R language. Bioinformatics 20, 289–290. (10.1093/bioinformatics/btg412) 14734327

[35] Harmon LJ, Weir JT, Brock CD, Glor RE, Challenger W 2008 GEIGER: investigating evolutionary radiations. Bioinformatics 24, 129–131. (10.1093/bioinformatics/btm538) 18006550

[36] R Development Core Team. 2013 R: a language and environment for statistical computing. Vienna, Austria: R Foundation for Statistical Computing (http://www.R-project.org/)

[37] Gilbert AN 1986 Mammary number and litter size in Rodentia: the ‘one half rule’. Proc. Natl Acad. Sci. USA 83, 4828–4830. (10.1073/pnas.83.13.4828) 16593720 PMC323835

[38] Diamond JM 1987 Arisotle's theory of mammalian teat number is confirmed. Nature 325, 200 (10.1038/325200a0) 3543685

[39] Sherman PW, Braude S, Jarvis JUM 1999 Litter sizes and mammary numbers of naked mole rats: breaking the one-half rule. J. Mammal. 80, 720–733. (10.2307/1383241)

[40] Orme CDL et al. 2012 CAPER: comparative analyses of phylogenetics and evolution in R. R package version 0.5.

[41] Lukas D, Clutton-Brock T 2012 Life histories and the evolution of cooperative breeding in mammals. Proc. R. Soc. B 279, 4065–4070. (10.1098/rspb.2012.1433) PMC3427589 22874752

[42] Pagel M, Meade A 2006 Bayesian analysis of correlated evolution of discrete characters by reversible-jump Markov chain Monte Carlo. Am. Nat. 167, 808–825. (10.1086/503444) 16685633

[43] Pagel M, Meade A, Barker D 2004 Bayesian estimation of ancestral character states on phylogenies. Syst. Biol. 53, 673–684. (10.1080/10635150490522232) 15545248

[44] Opie C, Atkinson QD, Dunbar RIM, Shultz S 2013 Male infanticide leads to social monogamy in primates. Proc. Natl Acad. Sci. USA 110, 13 328–13 332. (10.1073/pnas.1307903110) PMC3746880 23898180

[45] Rambaut A, Suchard MA, Xie D, Drummond AJ 2014 Tracer v1.6. See http://beast.bio.ed.ac.uk/Tracer.

[46] Pagel M 1999 Inferring the historical patterns of biological evolution. Nature 401, 877–884. (10.1038/44766) 10553904

[47] Komers PE, Brotherton PNM 1997 Female space use is the best predictor of monogamy in mammals. Proc. R. Soc. Lond. B 264, 1261–1270. (10.1098/rspb.1997.0174) PMC1688588 9332011

[48] Huck M, Fernandez-Duque E, Babb P, Schurr T 2014 Correlates of genetic monogamy in socially monogamous mammals: insights from Azara's owl monkeys. Proc. R. Soc. B 281, 20140195 (10.1098/rspb.2014.0195) PMC3973279 24648230

[49] Kokko H, Jennions MD 2008 Parental investment, sexual selection and sex ratios. J. Evol. Biol. 21, 919–948. (10.1111/j.1420-9101.2008.01540.x) 18462318

[50] Kvarnemo C 2006 Evolution and maintenance of male care: is increased paternity a neglected benefit of male care? Behav. Ecol. 17, 144–148. (10.1093/beheco/ari097)

[51] Hopwood PE, Moore AJ, Tregenza T, Royle NJ 2015 Male burying beetles extend, not reduce, parental care duration when reproductive competition is high. J. Evol. Biol. 28, 1394–1402. (10.1111/jeb.12664) 26033457

[52] Hardling R, Kaitala A 2004 Male brood care without paternity increases mating success. Behav. Ecol. 15, 715–721. (10.1093/beheco/arh046)

[53] Smuts BB, Gubernick DJ 1992 Male–infant relationships in non-human primates: paternal investment or mating effort? In Father–child relations (ed. Hewlett BS), pp. 1–30. New York, NY: Aldine de Gruyter.

[54] Buchan JC, Alberts SC, Silk JB, Altmann J 2003 True paternal care in a multi-male primate society. Nature 425, 179–181. (10.1038/nature01866) 12968180

[55] Bronson FH 1989 Mammalian reproductive biology. London, UK: University of Chicago Press.

[56] Speakman JR 2008 The physiological costs of reproduction in small mammals. Phil. Trans. R. Soc. B 363, 375–398. (10.1098/rstb.2007.2145) 17686735 PMC2606756

[57] Lack D 1947 The significance of clutch size. Ibis 89, 302–352. (10.1111/j.1474-919X.1947.tb04155.x)

[58] Williams GC 1966 Natural selection, the costs of reproduction and a refinement of Lack's principle. Am. Nat. 100, 687–690. (10.1086/282461)

[59] Dunbar RIM 1995 The mating system of callitrichid primates: I. Conditions for the coevolution of pair bonding and twinning. Anim. Behav. 50, 1057–1070. (10.1016/0003-3472(95)80106-5)

[60] Van Schaik C, Kappeler PM 2003 Evolution of social monogamy in primates. In Monogamy: mating strategies and partnerships in birds, humans, and other mammals (eds Reichard U, Boesh C), pp. 59–80. Cambridge, UK: Cambridge University Press.

[61] Ribble DO 2003 The evolution of social and reproductive monogamy in *Peromyscus*, evidence from *Peromyscus californicus* (the California Mouse). In Monogamy: mating strategies and partnerships in birds, humans, and other mammals (eds Reichard U, Boesh C), pp. 81–92. Cambridge, UK: Cambridge University Press.

[62] Trivers RL 1972 Parental investment and sexual selection. In Sexual selection and the descent of man, 1871–1971 (ed. Campbell B), pp. 136–179. Chicago, IL: Aldine.

[63] Parker GA, Royle NJ, Hartley IR 2002 Intrafamilial conflict and parental investment: a synthesis. Phil. Trans. R. Soc. Lond. B 357, 295–307. (10.1098/rstb.2001.0950) 11958698 PMC1692944

[64] McNamara JM, Houston AI, Barta Z, Osorno J-L 2003 Should young ever be better off with one parent than with two? Behav. Ecol. 14, 301–310. (10.1093/beheco/14.3.301)

[65] Royle NJ, Hartley IR, Parker GA 2002 Sexual conflict reduces offspring fitness in zebra finches. Nature 416, 733–736. (10.1038/416733a) 11961554

[66] Smith CC, Fretwell SD 1974 The optimal balance between size and number of offspring. Am. Nat. 108, 499–506. (10.1086/282929)

[67] Lukas D, Clutton-Brock TH 2011 Cooperative breeding and monogamy in mammalian societies. Proc. R. Soc. B 279, 2151–2156. (10.1098/rspb.2011.2468) PMC3321711 22279167

