# Supplementary material for: Paternal care and litter size coevolution in mammals
Source: Proc Biol Sci. 2016 Apr 27;283(1829):20160140. doi: 10.1098/rspb.2016.0140 (PMC4855383; doi:10.1098/rspb.2016.0140)
Supplement: Stockley&Hobson_Supplementary Materials [file rspb20160140supp1.docx]

**Paternal care and litter size coevolution in mammals**

**Paula Stockley and Liane Hobson**

**SUPPLEMENTARY MATERIALS**

**Table S1** Mammalian species included in the comparative analyses, including classifications based on presence or absence of male care, presence or absence of male care that includes provisioning, and sources of classifications. See main text for definitions of male care and provisioning.

| **Species** | **Male care** | **Male provisioning** | **Sources** |
| --- | --- | --- | --- |
| Afrosoricida |  |  |  |
| *Hemicentetes semispinosus* | yes | no | 1 |
| *Tenrec ecaudatus* | yes | yes | 1 |
| Artiodactyla |  |  |  |
| *Cephalophus callipygus* | no | no | 2 |
| *Cephalophus dorsalis* | no | no | 2 |
| *Cephalophus natalensis* | no | no | 2 |
| *Damaliscus lunatus* | no | no | 3 |
| *Hyemoschus aquaticus* | no | no | 2 |
| *Kobus vardonii* | no | no | 3 |
| *Madoqua kirkii* | no | no | 2 |
| *Neotragus batesi* | no | no | 2 |
| *Neotragus pygmaeus* | no | no | 2 |
| *Okapia johnstoni* | no | no | 2 |
| *Oreotragus oreotragus* | no | no | 2 |
| *Redunca arundinum* | no | no | 2 |
| *Redunca redunca* | no | no | 2 |
| Carnivora |  |  |  |
| *Acinonyx jubatus* | no | no | 1, 2 |
| *Ailuropoda melanoleuca* | no | no | 4 |
| *Ailurus fulgens* | no | no | 4 |
| *Alopex lagopus* | yes | yes | 1 |
| *Arctictis binturong* | no | no | 4 |
| *Atilax paludinosus* | no | no | 4 |
| *Bassariscus astutus* | yes | yes | 1, 2 |
| *Canis adustus* | yes |  | 4 |
| *Canis aureus* | yes |  | 4 |
| *Canis latrans* | yes | yes | 1 |
| *Canis lupus* | yes | yes | 1 |
| *Canis mesomelas* | yes | yes | 1 |
| *Caracal caracal* | no | no | 4 |
| *Cerdocyon thous* | yes | yes | 1, 2 |
| *Chrysocyon brachyurus* | yes |  | 4 |
| *Civettictis civetta* | no | no | 1, 2 |
| *Crocuta crocuta* | no | no | 1, 2 |
| *Cryptoprocta ferox* | no | no | 1, 2 |
| *Cuon alpinus* | yes | yes | 1 |
| *Enhydra lutris* | no | no | 1 |
| *Eupleres goudotii* | no | no | 1 |
| *Felis nigripes* | yes | yes | 1 |
| *Felis silvestris* | no | no | 1, 2 |
| *Galerella pulverulenta* | no | no | 1 |
| *Galerella sanguinea* | no | no | 1, 2 |
| *Galidia elegans* | yes | yes | 1, 2 |
| *Genetta genetta* | no | no | 1 |
| *Genetta tigrina* | no | no | 1 |
| *Gulo gulo* | no | no | 1 |
| *Helogale parvula* | yes | yes | 1, 2 |
| *Hyaena hyaena* | no | no | 4 |
| *Ichneumia albicauda* | no | no | 1, 2 |
| *Ictonyx striatus* | no | no | 1, 2 |
| *Leptailurus serval* | no | no | 1, 2 |
| *Lontra canadensis* | no | no | 1 |
| *Lutra lutra* | no | no | 1 |
| *Lutra maculicollis* | no | no | 1 |
| *Lutrogale perspicillata* | yes | yes | 1 |
| *Lycaon pictus* | yes | yes | 1, 2 |
| *Lynx lynx* | no | no | 1, 2 |
| *Lynx rufus* | no | no | 2 |
| *Martes americana* | no | no | 1 |
| *Martes martes* | yes | yes | 1 |
| *Martes pennanti* | no | no | 4 |
| *Meles meles* | no | no | 1, 2 |
| *Melursus ursinus* | no | no | 4 |
| *Mephitis mephitis* | no | no | 1 |
| *Mungos mungo* | yes | yes | 1 |
| *Mustela altaica* | no | no | 4 |
| *Mustela erminea* | no | no | 1 |
| *Mustela frenata* | no | no | 4 |
| *Mustela lutreola* | no | no | 4 |
| *Mustela nigripes* | no | no | 4 |
| *Mustela nivalis* | no | no | 1 |
| *Mustela putorius* | no | no | 4 |
| *Mustela sibirica* | no | no | 1 |
| *Mustela vison* | no | no | 2 |
| *Nandinia binotata* | no | no | 1, 2 |
| *Neofelis nebulosa* | yes | yes | 1 |
| *Nyctereutes procyonoides* | yes | yes | 1, 2 |
| *Otocyon megalotis* | yes | yes | 1, 2 |
| *Panthera leo* | yes | yes | 1, 2 |
| *Panthera onca* | no | no | 4 |
| *Panthera pardus* | no | no | 1, 2 |
| *Panthera tigris* | no | no | 4 |
| *Parahyaena brunnea* | yes | yes | 1, 2 |
| *Potos flavus* | no | no | 1 |
| *Prionailurus bengalensis* | yes | yes | 1, 5 |
| *Procyon lotor* | no | no | 1, 2 |
| *Proteles cristatus* | no | no | 1, 2 |
| *Puma concolor* | no | no | 1, 2 |
| *Pteromura brasiliensis* | yes | yes | 1 |
| *Speothos venaticus* | yes | yes | 1 |
| *Spilogale putorius* | no | no | 4 |
| *Suricata suricatta* | yes | no | 1 |
| *Taxidea taxus* | no | no | 4 |
| *Uncia uncia* | no | no | 1 |
| *Urocyon cinereoargenteus* | no | no | 4 |
| *Ursus americanus* | no | no | 1 |
| *Ursus arctos* | no | no | 1 |
| *Ursus maritimus* | no | no | 1, 2 |
| *Ursus thibetanus* | no | no | 4 |
| *Viverra zibetha* | no | no | 1 |
| *Vulpes velox* | yes | yes | 1 |
| *Vulpes vulpes* | yes | yes | 1, 2 |
| *Vulpes zerda* | yes | yes | 1 |
| Chiroptera |  |  |  |
| *Artibeus cinereus* | no | no | 6 |
| *Artibeus jamaicensis* | no | no | 6 |
| *Balantiopteryx plicata* | no | no | 6 |
| *Cardioderma cor* | no | no | 6 |
| *Carollia perspicillata* | no | no | 6 |
| *Coleura afra* | no | no | 6 |
| *Cynopterus brachyotis* | no | no | 6 |
| *Cynopterus horsfieldi* | no | no | 6 |
| *Cynopterus sphinx* | no | no | 6 |
| *Desmodus rotundus* | no | no | 6 |
| *Ectophylla alba* | no | no | 6 |
| *Hipposideros beatus* | no | no | 6 |
| *Hipposideros galeritus* | no | no | 6 |
| *Kerivoula lanosa* | no | no | 6 |
| *Kerivoula papillosa* | no | no | 6 |
| *Kerivoula picta* | no | no | 6 |
| *Lavia frons* | yes |  | 6 |
| *Macrotus californicus* | no | no | 6 |
| *Miniopterus australis* | no | no | 6 |
| *Miniopterus minor* | no | no | 6 |
| *Myotis adversus* | no | no | 6 |
| *Myotis bocagei* | no | no | 6 |
| *Myotis lucifugus* | no | no | 6 |
| *Myotis myotis* | no | no | 6 |
| *Noctilio leporinus* | no | no | 6 |
| *Nyctalus noctula* | no | no | 6 |
| *Nycteris arge* | no | no | 6 |
| *Nycteris hispida* | no | no | 6 |
| *Nycteris nana* | no | no | 6 |
| *Nycticeius humeralis* | no | no | 6 |
| *Otomops martiensseni* | no | no | 6 |
| *Peropteryx kappleri* | no | no | 6 |
| *Phyllostomus discolor* | yes | no | 1 |
| *Phyllostomus hastatus* | no | no | 6 |
| *Pipistrellus nanus* | no | no | 6 |
| *Pipistrellus nathusii* | no | no | 6 |
| *Pipistrellus pipistrellus* | no | no | 6 |
| *Plecotus auritus* | no | no | 6 |
| *Pteropus hypomelanus* | no | no | 6 |
| *Pteropus mariannus* | no | no | 6 |
| *Pteropus pumilus* | no | no | 6 |
| *Pteropus rodricensis* | no | no | 6 |
| *Pteropus samoensis* | no | no | 6 |
| *Pteropus seychellensis* | no | no | 6 |
| *Pteropus tonganus* | no | no | 6 |
| *Pteropus vampyrus* | no | no | 6 |
| *Rhinolophus ferrumequinum* | no | no | 6 |
| *Rhinolophus luctus* | no | no | 6 |
| *Rhinolophus sedulus* | no | no | 6 |
| *Rhynchonycteris naso* | no | no | 6 |
| *Rousettus amplexicaudatus* | yes | no | 1 |
| *Saccolaimus peli* | no | no | 6 |
| *Saccopteryx leptura* | no | no | 6 |
| *Tadarida brasiliensis* | no | no | 6 |
| *Tylonycteris pachypus* | no | no | 6 |
| *Tylonycteris robustula* | no | no | 6 |
| *Uroderma bilobatum* | no | no | 6 |
| *Vampyressa nymphaea* | no | no | 6 |
| *Vampyrum spectrum* | yes | yes | 1, 6 |
| Diprotodontia |  |  |  |
| *Aepyprymnus rufescens* | no | no | 7 |
| *Antechinomys laniger* | no | no | 7 |
| *Antechinus flavipes* | no | no | 7 |
| *Antechinus minimus* | no | no | 7 |
| *Antechinus stuartii* | no | no | 7 |
| *Antechinus swainsonii* | no | no | 7 |
| *Bettongia gaimardi* | no | no | 7 |
| *Bettongia lesueur* | no | no | 7 |
| *Bettongia penicillata* | no | no | 7 |
| *Bettongia tropica* | no | no | 7 |
| *Burramys parvus* | no | no | 7 |
| *Caluromys derbianus* | no | no | 7 |
| *Caluromys philander* | no | no | 7 |
| *Cercartetus caudatus* | no | no | 7 |
| *Cercartetus nanus* | no | no | 7 |
| *Dactylopsila trivirgata* | no | no | 7 |
| *Dasycercus cristicauda* | no | no | 7 |
| *Dasykaluta rosamondae* | no | no | 7 |
| *Dasyuroides byrnei* | no | no | 7 |
| *Dasyurus geoffroii* | no | no | 7 |
| *Dasyurus hallucatus* | no | no | 7 |
| *Dasyurus viverrinus* | no | no | 7 |
| *Dendrolagus bennettianus* | no | no | 7 |
| *Dendrolagus lumholtzi* | no | no | 7 |
| *Didelphis marsupialis* | no | no | 7 |
| *Didelphis virginiana* | no | no | 7 |
| *Dorcopsis muelleri* | no | no | 7 |
| *Dromiciops gliroides* | no | no | 7 |
| *Echymipera rufescens* | no | no | 7 |
| *Isoodon macrourus* | no | no | 7 |
| *Isoodon obesulus* | no | no | 7 |
| *Lagorchestes conspicillatus* | no | no | 7 |
| *Lagorchestes hirsutus* | no | no | 7 |
| *Lagostrophus fasciatus* | no | no | 7 |
| *Lasiorhinus latifrons* | no | no | 7 |
| *Macropus agilis* | no | no | 7 |
| *Macropus antilopinus* | no | no | 7 |
| *Macropus dorsalis* | no | no | 7 |
| *Macropus eugenii* | no | no | 7 |
| *Macropus fuliginosus* | no | no | 7 |
| *Macropus giganteus* | no | no | 7 |
| *Macropus irma* | no | no | 7 |
| *Macropus parma* | no | no | 7 |
| *Macropus parryi* | no | no | 7 |
| *Macropus robustus* | no | no | 7 |
| *Macropus rufogriseus* | no | no | 7 |
| *Macropus rufus* | no | no | 7 |
| *Macrotis lagotis* | no | no | 7 |
| *Myrmecobius fasciatus* | no | no | 7 |
| *Ningaui ridei* | no | no | 7 |
| *Ningaui yvonnae* | no | no | 7 |
| *Notoryctes typhlops* | no | no | 7 |
| *Onychogalea fraenata* | no | no | 7 |
| *Onychogalea unguifera* | no | no | 7 |
| *Parantechinus apicalis* | no | no | 7 |
| *Perameles bougainville* | no | no | 7 |
| *Perameles gunnii* | no | no | 7 |
| *Perameles nasuta* | no | no | 7 |
| *Petaurus breviceps* | yes | no | 8, 9 |
| *Petaurus gracilis* | yes |  | 10 |
| *Petropseudes dahli* | yes | no | 11 |
| *Petrogale assimilis* | no | no | 7 |
| *Petrogale concinna* | no | no | 7 |
| *Petrogale penicillata* | no | no | 7 |
| *Petrogale xanthopus* | no | no | 7 |
| *Phalanger mimicus* | no | no | 7 |
| *Phascogale calura* | no | no | 7 |
| *Phascogale tapoatafa* | no | no | 7 |
| *Phascolarctos cinereus* | no | no | 7 |
| *Philander opossum* | no | no | 7 |
| *Planigale gilesi* | no | no | 7 |
| *Planigale ingrami* | no | no | 7 |
| *Planigale maculata* | no | no | 7 |
| *Planigale tenuirostris* | no | no | 7 |
| *Potorous tridactylus* | no | no | 7 |
| *Pseudantechinus bilarni* | no | no | 7 |
| *Pseudantechinus macdonnellensis* | no | no | 7 |
| *Sarcophilus harrisii* | no | no | 7 |
| *Setonix brachyurus* | no | no | 7 |
| *Sminthopsis griseoventer* | no | no | 7 |
| *Sminthopsis leucopus* | no | no | 7 |
| *Spilocuscus maculatus* | no | no | 7 |
| *Tarsipes rostratus* | no | no | 7 |
| *Thylogale billardierii* | no | no | 7 |
| *Thylogale stigmatica* | no | no | 7 |
| *Thylogale thetis* | no | no | 7 |
| *Trachypithecus pileatus* | no | no | 7 |
| *Trichosurus caninus* | no | no | 7 |
| *Trichosurus vulpecula* | no | no | 7 |
| *Vombatus ursinus* | no | no | 7 |
| *Wallabia bicolor* | no | no | 7 |
| *Wyulda squamicaudata* | no | no | 7 |
| Lagomorpha |  |  |  |
| *Lepus timidus* | no | no | 2 |
| *Ochotona curzoniae* | no | no | 2 |
| *Oryctolagus cuniculus* | no | no | 1 |
| *Sylvilagus aquaticus* | no | no | 2 |
| *Sylvilagus floridanus* | no | no | 2 |
| Macroscelidea |  |  |  |
| *Elephantulus intufi* | no | no | 12 |
| *Elephantulus rufescens* | yes | no | 12 |
| *Macroscelides proboscideus* | no | no | 2 |
| *Rhynchocyon chrysopygus* | no | no | 2 |
| Primates |  |  |  |
| *Alouatta palliata* | no | no | 13 |
| *Alouatta pigra* | yes | no | 14 |
| *Alouatta seniculus* | no | no | 5 |
| *Aotus azarai* | yes | yes | 14,15 |
| *Aotus nancymaae* | yes | yes | 16 |
| *Aotus trivirgatus* | yes | yes | 1, 2, 5, 15 |
| *Ateles fusciceps* | yes |  | 13 |
| *Ateles geoffroyi* | yes |  | 13 |
| *Callicebus cupreus* | yes |  | 17 |
| *Callicebus moloch* | yes | yes | 1, 2, 5, 14 |
| *Callicebus torquatus* | yes | yes | 5 |
| *Callimico goeldii* | yes | yes | 14,17 |
| *Callithrix jacchus* | yes | yes | 1, 2, 5 |
| *Callithrix pygmaea* | yes | no | 1, 2, 5 |
| *Cebus albifrons* | yes | no | 1, 2, 5 |
| *Cebus apella* | yes | no | 14 |
| *Cercopithecus ascanius* | no | no | 13 |
| *Cercopithecus mitis* | no | no | 13 |
| *Cercopithecus neglectus* | no | no | 2, 5 |
| *Cheirogaleus medius* | yes | no | 14, 18, 19 |
| *Chlorocebus aethiops* | no | no | 13 |
| *Colobus polykomos* | no | no | 13 |
| *Erythrocebus patas* | no | no | 1, 2 |
| *Eulemur fulvus* | no | no | 5, 14 |
| *Eulemur macaco* | no | no | 2, 5 |
| *Eulemur mongoz* | yes | no | 20 |
| *Galago senegalensis* | no | no | 14 |
| *Gorilla gorilla* | no | no | 13 |
| *Hapalemur griseus* | yes | no | 2, 14 |
| *Homo sapiens* | yes | yes | 5, 14 |
| *Hylobates agilis* | no | no | 21 |
| *Hylobates concolor* | no | no | 21 |
| *Hylobates gabriellae* | no | no | 21 |
| *Hylobates hoolock* | no | no | 21 |
| *Hylobates klossii* | no | no | 21 |
| *Hylobates lar* | no | no | 21 |
| *Hylobates leucogenys* | no | no | 21 |
| *Hylobates moloch* | no | no | 21 |
| *Hylobates muelleri* | no | no | 21 |
| *Hylobates pileatus* | no | no | 21 |
| *Hylobates syndactylus* | yes | yes | 21 |
| *Leontopithecus rosalia* | yes | yes | 1, 2, 5 |
| *Lepilemur ruficaudatus* | no | no | 22 |
| *Lophocebus albigena* | yes | no | 5 |
| *Macaca arctoides* | yes | yes | 1, 2, 5, 13 |
| *Macaca fascicularis* | no | no | 5 |
| *Macaca fuscata* | no | no | 13 |
| *Macaca mulatta* | no | no | 13 |
| *Macaca nemestrina* | no | no | 1, 2, 5, 14 |
| *Macaca radiata* | no | no | 13 |
| *Macaca sylvanus* | yes | no | 1, 5, 14 |
| *Macaca thibetana* | yes |  | 23 |
| *Miopithecus talapoin* | no | no | 13 |
| *Nasalis concolor* | no | no | 5 |
| *Pan troglodytes* | no | no | 13, 14 |
| *Papio anubis* | yes | yes | 1, 2, 5 |
| *Pithecia pithecia* | yes |  | 13 |
| *Pongo pygmaeus* | no | no | 1, 2, 14 |
| *Presbytis melalophos* | no | no | 5 |
| *Presbytis potenziani* | no | no | 5 |
| *Propithecus verreauxi* | no | no | 14 |
| *Pygathrix bieti* | yes | no | 24 |
| *Saguinus fuscicollis* | yes | yes | 1, 2, 5, 14 |
| *Saguinus labiatus* | yes |  | 13 |
| *Saguinus nigricollis* | no | no | 5 |
| *Saguinus oedipus* | yes | yes | 1, 2, 5 |
| *Saimiri sciureus* | no | no | 2, 5, 14 |
| *Semnopithecus entellus* | no | no | 1, 5 |
| *Tarsius bancanus* | no | no | 25 |
| *Tarsius syrichta* | yes | no | 1 |
| *Theropithecus gelada* | no | no | 13 |
| *Trachypithecus johnii* | no | no | 1 |
| *Trachypithecus obscurus* | no | no | 5 |
| *Varecia variegata* | no | no | 5 |
| Rodentia |  |  |  |
| *Acomys cahirinus* | yes | no | 26 |
| *Agouti paca* | yes | no | 1, 27 |
| *Akodon azarae* | no | no | 28 |
| *Apodemus sylvaticus* | no | no | 5 |
| *Baiomys taylori* | yes | no | 1, 26 |
| *Calomys laucha* | yes | no | 28, 29 |
| *Calomys musculinus* | no | no | 28, 29 |
| *Castor canadensis* | yes | yes | 2, 26 |
| *Castor fiber* | yes | yes | 1, 26 |
| *Cavia aperea* | no | no | 30 |
| *Chinchilla lanigera* | yes | no | 1 |
| *Clethrionomys gapperi* | no | no | 31 |
| *Clethrionomys glareolus* | no | no | 32 |
| *Clethrionomys rutilus* | no | no | 31 |
| *Coendou prehensilis* | no | no | 2 |
| *Cricetulus migratorius* | no | no | 31 |
| *Dasyprocta punctata* | yes | no | 26 |
| *Dicrostonyx richardsoni* | yes | no | 33,34 |
| *Dolichotis patagonum* | yes | no | 1, 26 |
| *Erethizon dorsatum* | no | no | 2 |
| *Galea musteloides* | no | no | 30 |
| *Galea spixii* | no | no | 30 |
| *Heterocephalus glaber* | yes | yes | 1, 26 |
| *Hystrix africaeaustralis* | yes | no | 1, 2, 26 |
| *Kannabateomys amblyonyx* | yes | yes | 35 |
| *Kerodon rupestris* | yes | no | 26, 30 |
| *Lasiopodomys brandtii* | yes | no | 31 |
| *Lasiopodomys mandarinus* | yes | no | 36 |
| *Marmota caligata* | no | no | 26 |
| *Meriones crassus* | no | no | 1, 26 |
| *Meriones tamariscinus* | yes | no | 1, 26 |
| *Meriones unguiculatus* | yes | no | 1, 2, 26 |
| *Mesocricetus auratus* | no | no | 27, 31 |
| *Microcavia australis* | no | no | 2, 26 |
| *Microtus agrestis* | no | no | 37 |
| *Microtus californicus* | yes | no | 1, 2, 26 |
| *Microtus montanus* | no | no | 37, 38 |
| *Microtus ochrogaster* | yes | no | 1, 2, 26 |
| *Microtus oeconomus* | yes | no | 39 |
| *Microtus pennsylvanicus* | no | no | 27, 37 |
| *Microtus pinetorum* | yes |  | 37 |
| *Microtus richardsoni* | no | no | 37 |
| *Microtus xanthognathus* | no | no | 37 |
| *Mus musculus* | no | no | 40 |
| *Mus spicilegus* | yes | no | 41 |
| *Mus spretus* | yes | no | 42 |
| *Myoprocta acouchy* | yes | no | 1, 26 |
| *Neotoma albigula* | no | no | 27 |
| *Neotoma floridana* | no | no | 2 |
| *Neotoma fuscipes* | no | no | 2 |
| *Neotoma lepida* | no | no | 2 |
| *Neotoma micropus* | no | no | 27 |
| *Neotomodon alstoni* | yes | no | 43 |
| *Notomys alexis* | yes | no | 1, 26 |
| *Octodon degus* | yes | no | 1, 26 |
| *Octodontomys gliroides* | yes | no | 1, 26 |
| *Ondatra zibethicus* | yes | yes | 44 |
| *Onychomys leucogaster* | yes | yes | 1, 2, 26 |
| *Onychomys torridus* | yes | no | 1, 26 |
| *Peromyscus boylii* | no | no | 27 |
| *Peromyscus californicus* | yes | no | 1, 2, 26 |
| *Peromyscus crinitus* | no | no | 27 |
| *Peromyscus eremicus* | yes | no | 26 |
| *Peromyscus leucopus* | yes | no | 1, 2, 26 |
| *Peromyscus maniculatus* | yes | no | 1, 2, 26 |
| *Peromyscus melanocarpus* | yes | no | 1, 26 |
| *Peromyscus melanophrys* | no | no | 45 |
| *Peromyscus mexicanus* | yes | no | 1, 26 |
| *Peromyscus polionotus* | yes | no | 1, 26 |
| *Peromyscus truei* | no | no | 27 |
| *Petromus typicus* | yes | no | 46 |
| *Phodopus campbelli* | yes | yes | 47 |
| *Phodopus sungorus* | no | no | 27 |
| *Pseudomys albocinereus* | yes | no | 1, 26 |
| *Pseudomys desertor* | no | no | 26 |
| *Rattus fuscipes* | yes | no | 1, 26 |
| *Rattus norvegicus* | no | no | 27 |
| *Rattus rattus* | yes | no | 27 |
| *Reithrodontomys humulis* | yes | no | 1, 26 |
| *Rhabdomys pumilio* | yes | no | 48 |
| *Sciurus carolinensis* | no | no | 2 |
| *Sciurus niger* | no | no | 2 |
| *Sigmodon hispidus* | no | no | 27 |
| *Spermophilus parryii* | no | no | 26 |
| *Tamias striatus* | no | no | 2 |
| *Tamiasciurus hudsonicus* | no | no | 2 |
| Soricomorpha |  |  |  |
| *Crocidura russula* | yes | no | 49, 50 |
| *Cryptotis parva* | yes | no | 1 |
| *Sorex araneus* | no | no | 2 |
| *Sorex minutus* | no | no | 2 |
| *Sorex unguiculatus* | no | no | 2 |
| *Suncus etruscus* | yes | no | 1 |

**Sources**

1. Kleiman, D. G. & Malcolm J. R. 1981 The evolution of male parental investment. In *Parental care in mammals* (ed. D. J. Gubernick & P. H. Klopfer), pp. 347-387. New York & London: Plenum.

2. Komers, P. E. & Brotherton, P. N. M. 1997 Female space use is the best predictor of monogamy in mammals. *Proc. R. Soc. Lond. B* **264,** 1261-1270.

3. Balmford, A., Rosser, A. M. & Albon, S. D. 1992 Correlates of female choice in resource-defending antelope. *Behav. Ecol. Sociobiol.* **31,** 107-114.

4. Gittleman, J. L. 1994 Female brain size and parental care in carnivores. *Proc. Natl. Acad. Sci.* USA **91,** 5495-5497.

5. Nowak, R. M. 1999 *Walker’s mammals of the world*. Baltimore: The Johns Hopkins University Press.

6. McCracken, G. F. & Wilkinson, G. S. 2000 Bat mating systems. In *Reproductive biology of bats*. E. G. Crichton & P. H. Krutzsch (eds.), Academic Press, pp. 321-362.

7. Weisbecker, V., Blomberg, S., Goldzien, A. W., Brown, M. & Fisher, D. 2015 The evolution of relative brain size in marsupials is energetically constrained but not driven by behavioural complexity. *Brain Behav. Evol.* **85,** 125-135.

8. Klettenheimer, B. S., TempleSmith, P. D. & Sofronidis, G. 1997 Father and son sugar gliders: more than a genetic coalition? *J. Zool.* **242,** 741-750.

9. Goldingay, R. 2010 Direct male care observed in wild sugar gliders. *Aust. Mammal*. **32,** 177-178.

10. Muller, T. L., Ensabella, T. J., Booth, R., Johnston, S. D. & Phillips, C. J. C. 2010 The behaviour and environmental enrichment of captive mahogany gliders (*Petaurus gracilis*). *Aust. Mammal.* **32,** 109-116.

11. Runcie, M. J. 2000 Biparental care and obligate monogamy in the rock-haunting possum, *Petropseudes dahlia*, from tropical Australia. *Anim. Behav*. **59,** 1001-1008.

12. Rathbun, G. B. & Rathbun, C. D. 2006 Social structure of the bushveld sengi (*Elephantulus intufi*) in Namibia and the evolution of monogamy in the Macroscelidea. *J. Zool.* **269,** 391-399.

13. Ross, C. & MacLarnon, A. 2000 The evolution of non-maternal care in anthropoid primates: a test of the hypotheses. *Folia Primatol*. **71,** 93-113.

14. Muller, M. & Emery Thompson, M. 2012 Mating, parenting and male reproductive strategies. In *The evolution of primate societies*. J. C. Mitani, J. Call, P. M. Kappeler, R. A. Palombit & J. B. Silk (eds.), University of Chicago Press, Chicago, pp. 387-411.

15. Wolovich, C. K., Perea-Rodrigeuz, J. B. & Fernandez-Duque, E. 2008 Food transfers to young and mates in wild owl monkeys (*Aotus azarai*). *Am. J. Primatol.* **70,** 211-221.

16. Wolovich, C. K., Evans, S. & Green, S. M. 2010 Mated pairs of owl monkeys (*Aotus nancymaae*) exhibit sex differences in response to unfamiliar male and female conspecifics. *Am. J. Primatol.* **72,** 942-950.

17. Schradin, C., Reeder, D. A. M., Mendoza, S. P. & Anzenberger, G. 2003 Prolactin and paternal care: comparison of three species of monogamous New World monkeys (*Callicebus cupreus, Callithrix jacchus*, and *Callimico goeldii*). *J. Comp. Psychol*. **117,** 166-175.

18. Fietz, J. 1999 Monogamy as a rule rather than exception in nocturnal lemurs: the case of the fat-tailed dwarf lemur, *Cheirogaleus medius*. *Ethology* **105,** 259-272.

19. Fietz, J. & Dausmann, K. H. 2003 Costs and potential benefits of parental care in the nocturnal fat-tailed dwarf lemur (*Cheirogaleus medius*). *Folia Primatol*. **74,** 246-258.

20. Curtis, D. J. & Zaramody, A. 1999 Social structure and seasonal variation in the behaviour of *Eulemur mongoz*. *Folia Primatol.* **70,** 79-96.

21. Rafacz, M. L., Margulis, S. & Santymire, R. M. 2012 Hormonal correlates of paternal care differences in the Hylobatidae. *Am. J. Primatol.* **74,** 247-260.

22. Hilgartner, R., Fitchtel, C., Kappeler, P. M. & Zinner, D. 2012 Determinants of pair-living in red-tailed sportive lemurs (*Lepilemur ruficaudatus*). *Ethology* **118,** 466-479.

23. Deng, Z. Y. 1993 Social development of infants of *Macaca thibetana* at Mount Emei, China. *Folia Primatol.* **60,** 28-35.

24. Xiang, Z. F., Sayers, K. & Grueter, C. C. 2009 Direct paternal care in black-and-white snub-nosed monkeys. *J. Zool.* **278,** 157-162.

25. Roberts, M. 1994 Growth, development, and parental care in the Western Tarsier (*Tarsius bancanus*) in captivity: evidence for a “slow” life history and non-monogamous mating system. *Int. J. Primatol.* **15,** 1-28.

26. Dewsbury, D. A. 1985 Paternal behaviour in rodents. *Amer. Zool.* **25,** 841-852.

27. Lonstein, J. S. & De Vries, G. J. 2000 Sex differences in the parental behaviour of rodents.

28. Laconi, M. R. & Castro-Vazquez, A. 1999 Nest building and parental behaviour in two species of *Calomys* (Muridae, Sigmodontidae): a laboratory study. *Mammalia* **63,** 11-20.

29. Laconi, M. R., Jahn, G. A. & Castro-Vazquez, A. 2000 Influence of different social partners on the survival and growth of pups in two species of *Calomys* (Muridae, Sigmodontinae). *Ethol. Ecol. Evol.* **12,** 187-196.

30. Adrian, O. & Sachser, N. 2011 Diversity of social and mating systems in cavies: a review. *J. Mammal.* **92,** 39-53.

31. Gromov, V. S. 2011 Pair-bonding and parental care in cricetid rodents: a comparative study. *Acta Theriol.* **56,** 23-33.

32. Classification based on authors’ experience of breeding *Myodes glareolus* under laboratory conditions.

33. Shilton, C. M. & Brooks, R. J. 1989 Paternal care in captive collared lemmings (*Dicrostonyx richardsoni*) and its effect on development of the offspring. *Can. J. Zool.* **67,** 2740-2745.

34. Gajda, A. & Brooks, R. J. 1993 Paternal care in collared lemmings (*Dicrostonyx richardsoni*) –artefact or adaptation. *Arctic* **46,** 312-315.

35. Silva, R. B., Vieira, E. M. & Izar, P. 2008 Social monogamy and biparental care of the neotropical southern bamboo rat (*Kannabateomys amblyonyx*). *J. Mammal.* **89,** 1464-1472.

36. Smorkatcheva, A. 2003 Parental care in the captive mandarin vole, *Lasiopodomys mandarinus*. *Canadian Journal of Zoology* **81,** 1339-1345.

37. Wang, Z. & Insel, T. R. 1996 Parental behavior in voles. *Adv. Study Behav.* **25,** 361-384; Parental Care: Evolution, Mechanisms, and Adaptive Significance (eds J. S. Rosenblatt & C. T. Snowden)

38. McGuire, B. & Novak, M. 1986 Parental care and its relationship to social organisation in the montane vole (*Microtus montanus*). *J. Mammal.* **67,** 305-311.

39. Ims, R. A. 1997 Determinants of geographic variation in growth and reproductive traits in the root vole. *Ecology* **78,** 461-470.

40. Male house mice do show some paternal behaviour (see refs 26, 41); however as this is facultative and highly variable between individuals, with more than 50% of individuals tested showing no paternal behaviour (41, and authors’ unpublished data), we have here classed *Mus musculus* conservatively as not having paternal care.

41. Patris, B. & Baudoin, C. 2000 A comparative study of parental care between two rodent species: implications for the mating system of the mound-building mouse, *Mus spicilegus*. *Behavioural Processes* **51,** 35-43.

42. Cassaing, J., Cervera, S. & Isaac, F. 2010 Laboratory and field evidence of paternal care in the Algerian mouse (*Mus spretus*). *J. Ethol.* **28,** 7-13.

43. Luis, J., Ramirez, L., Carmona, A., Ortiz, G., Delgado, J. & Cardenos, R. 2009 Paternal behavior and testosterone plasma levels in the volcano mouse, *Neotomodon alstoni* (Rodentia: Muridae). *Rev. Biol. Trop.* **57,** 433-439.

44. Marinelli, L., Messier, F. & Plante, Y. 1997 Consequences of following a mixed reproductive strategy in muskrats. *J. Mammal*. **78,** 163-172.

45. Ferkin, M. H. 1987 Parental care and social interactions of captive plateau mice, *Peromyscus melanophrys*. *J. Mammal.* **68,** 266-274.

46. Rathbun, G. B. & Rathbun, C. D. 2006 Social monogamy in the noki or dassie-rat (*Petromus typicus*) in Namibia. *Mamm. Biol.* **71,** 203-213.

47. Wynne-Edwards, K. E. 1995 Biparental care in Djungarian but not Siberian dwarf hamsters. *Anim. Behav.* **50,** 1571-1585.

48. Schradin, C. & Pillay, N. 2003 Paternal care in the social and diurnal striped mouse (*Rhabdomys pumilio*): Laboratory and field evidence. *J. Comp. Psychol.* **117,** 317-324.

49. Vogel, P. 1969 Observations on the intraspecific behaviour of the shrew *Crocidura russula*. *Revue Suisse de Zoologie* **76,** 1079-1086.

50. Cantoni, D. & Vogel, P. 1989 Social organisation and mating system of free ranging, greater white-toothed shrews, *Crocidura russula*. *Anim. Behav.* **38,** 205-214.

**Table S2.** Most frequently observed models for coevolution between male care with provisioning and large litter size relative to teat number in mammals, using a Markov Chain Monte Carlo (MCMC) sampling algorithm and reversible jump (RJ) procedure in BayesTraits (see main text for further details). The table shows the top 10 and 50^th^ most frequently observed models of evolution, in the post-convergence portion of the run with the median likelihood value. Values in the model string represent each transition rate in the dependent model (trait order: q12, q13, q21, q24, q31, q34, q42, q43). A value of “Z” for a given transition rate indicates that it is predicted to be zero (i.e. the transition is not predicted to occur). Transition rates represented by the same numerical value are predicted to be not significantly different from one another. The top 10 most frequently observed models are models of correlated / dependent evolution. This is consistent with results of maximum likelihood tests reported in the main text.

| **Rank** | **Model string** | **Frequency** |
| --- | --- | --- |
| 1 | 'Z 1 1 Z 1 0 2 0 | 174 |
| 2 | 'Z 1 1 Z 1 0 0 1 | 93 |
| 3 | 'Z 1 1 Z 1 0 0 Z | 79 |
| 4 | 'Z 1 1 Z 2 0 2 0 | 56 |
| 5 | 'Z 1 1 Z Z 0 0 Z | 54 |
| 6 | 'Z 1 1 Z Z 0 2 0 | 50 |
| 7 | 'Z 1 1 Z Z 0 0 1 | 42 |
| 8 | 'Z 0 0 Z 0 1 1 Z | 36 |
| 9 | 'Z 1 1 1 1 0 0 1 | 35 |
| 10 | 'Z 0 0 Z 0 1 1 0 | 32 |
| 50 | 'Z 0 0 0 0 1 1 1 | 1 |

**Table S3.** Phylogenetically controlled PGLS models of the relationships between paternal care and offspring production in polytocous mammals, with cooperatively breeding species excluded (see main text for further details). Models test for relationships with average: a) litter size and b) offspring number per teat, linked to paternal care that includes provisioning of offspring. Body mass is included as a covariate in both models. Significant values (P<0.05) are presented in bold text. For the phylogenetic scaling parameter **λ**, superscripts indicate if values are significantly different from 0 or 1 respectively (where ns = not significantly different, and * = significantly different at P<0.05) in likelihood ratio tests.

| **Trait** | **λ** | **d.f.** | **Predictor** | **Slope ± SE** | ***t*** | ***P*** |
| --- | --- | --- | --- | --- | --- | --- |
| a) Litter size | 0.92^*,*^ | 207 | Body mass | -0.03±0.02 | -1.96 | 0.051 |
|  |  |  | Paternal provisioning | **0.05±0.02** | **2.16** | **0.048** |
|  |  |  |  |  |  |  |
| b) Offspring per teat | 0.69^*,*^ | 131 | Body mass | -0.04±0.02 | -1.74 | 0.084 |
|  |  |  | Paternal provisioning | 0.10±0.05 | 1.92 | 0.056 |
|  |  |  |  |  |  |  |


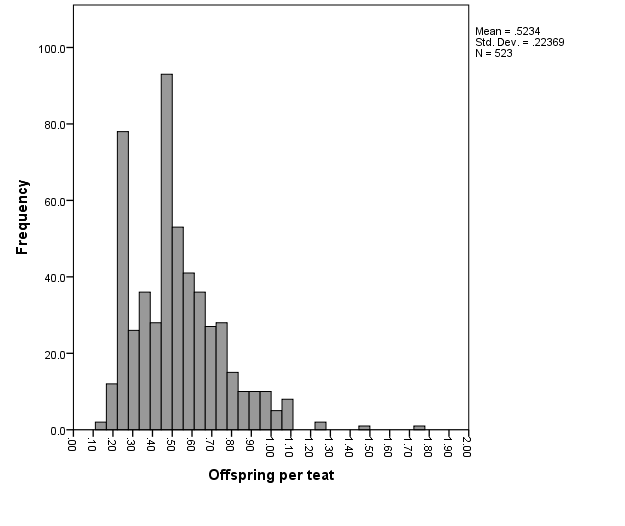


**Figure S1.** Distribution of offspring to teat ratios for all mammalian species with data available in Jones et al. (2009) (ref 30 in main text), including both polytocous and monotocous mammals (n=523). Consistent with the ‘one half rule’ for mammals, the median offspring to teat ratio is 0.50.


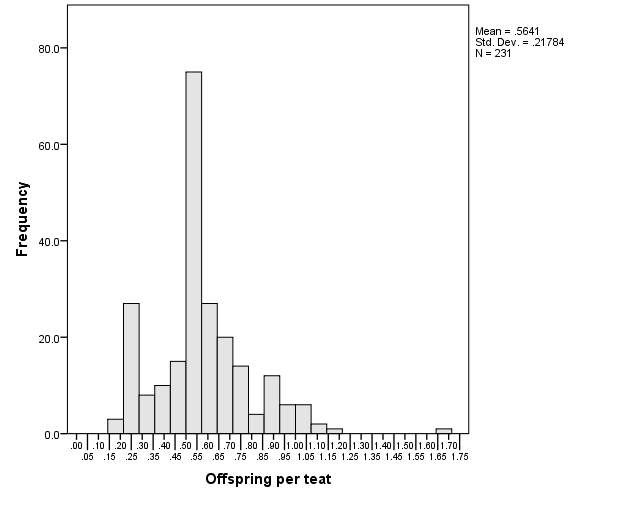


**Figure S2.** Distribution of offspring to teat ratios for mammalian species included in the main dataset (Table S1). For analyses based on binary traits, species were split according to whether offspring per teat ratios are relatively high (greater than or equal to 0.55) or relatively low (less than 0.55). The red dotted line on the figure indicates where this division falls within the overall distribution of ratios. The median offspring to teat ratio for species in this dataset is 0.51 (n=230). Species classed as having relatively high offspring to teat ratios (n=118) were found in diverse orders, including Afrosoricida, Carnivora, Diprodontia, Lagomorpha, Macroscelidea, Primates, Rodentia, and Soricomorpha, and were not found exclusively among mammalian lineages where male provisioning occurs (Afrosoricida, Carnivora, Primates, Rodentia, Chiroptera). Among species with relatively high offspring to teat ratios, 16% (n=19) were also classed as having paternal care that includes male provisioning (the full dataset is available in Dryad).
